# Macrophage IL-1β-positive microvesicles exhibit thrombo-inflammatory properties and are detectable in patients with active juvenile idiopathic arthritis

**DOI:** 10.3389/fimmu.2023.1228122

**Published:** 2023-11-21

**Authors:** Audrey Cambon, Charlotte Rebelle, Richard Bachelier, Laurent Arnaud, Stéphane Robert, Marie Lagarde, Romain Muller, Edwige Tellier, Yéter Kara, Aurélie Leroyer, Catherine Farnarier, Loris Vallier, Corinne Chareyre, Karine Retornaz, Anne-Laure Jurquet, Tu-Anh Tran, Romaric Lacroix, Françoise Dignat-George, Gilles Kaplanski

**Affiliations:** ^1^ Aix-Marseille University, Institut National de la Santé Et de la Recherche Médicale (INSERM), Institut National de la Recherche pour l’Agriculture et l’Environnement (INRAE), Centre de Recherche en CardioVasculaire et Nutrition (C2VN), Marseille, France; ^2^ Service de Médecine interne et d’Infectiologie, Hôpital d’Instruction des Armées (HIA) Sainte-Anne, Service de Santé des Armées (SSA), Toulon, France; ^3^ Service de Pédiatrie, Assistance Publique des Hôpitaux de Marseille (AP-HM), Hôpital Nord, Marseille, France; ^4^ Laboratoire d’Hématologie, Assistance Publique des Hôpitaux de Marseille (AP-HM), La Timone, Marseille, France; ^5^ Service de Médecine interne et d’Immunologie clinique, Assistance Publique des Hôpitaux de Marseille (AP-HM), La Conception, Marseille, France; ^6^ Laboratoire d’Immunologie, Assistance Publique des Hôpitaux de Marseille (AP-HM), La Conception, Marseille, France; ^7^ Service de Pédiatrie, Centre Hospitalo-Universitaire (CHU) Nîmes, Hôpital Carémeau, Nîmes, France

**Keywords:** microvesicles, IL-1β, NLRP3-inflammasome, tissue factor, juvenile idiopathic arthritis

## Abstract

**Objective:**

IL-1β is a leaderless cytokine with poorly known secretory mechanisms that is barely detectable in serum of patients, including those with an IL-1β-mediated disease such as systemic juvenile idiopathic arthritis (sJIA). Leukocyte microvesicles (MVs) may be a mechanism of IL-1β secretion. The first objective of our study was to characterize IL-1β-positive MVs obtained from macrophage cell culture supernatants and to investigate their biological functions *in vitro* and *in vivo*. The second objective was to detect circulating IL-1β-positive MVs in JIA patients.

**Methods:**

MVs were purified by serial centrifugations from PBMCs, or THP-1 differentiated into macrophages, then stimulated with LPS ± ATP. MV content was analyzed for the presence of IL-1β, NLRP3 inflammasome, caspase-1, P2X7 receptor, and tissue factor (TF) using ELISA, Western blot, or flow cytometry. MV biological properties were studied *in vitro* by measuring VCAM-1, ICAM-1, and E-selectin expression after HUVEC co-culture and factor-Xa generation test was realized. *In vivo*, MVs’ ability to recruit leukocytes in a murine model of peritonitis was evaluated. Plasmatic IL-1β-positive MVs were studied *ex vivo* in 10 active JIA patients using flow cytometry.

**Results:**

THP-1-derived macrophages stimulated with LPS and ATP released MVs, which contained NLRP3, caspase-1, and the 33-kDa precursor and 17-kDa mature forms of IL-1β and bioactive TF. IL-1β-positive MVs expressed P2X7 receptor and released soluble IL-1β in response to ATP stimulation *in vitro*. In mice, MVs induced a leukocyte peritoneal infiltrate, which was reduced by treatment with the IL-1 receptor antagonist. Finally, IL-1β-positive MVs were detectable in plasma from 10 active JIA patients.

**Conclusion:**

MVs shed from activated macrophages contain IL-1β, NLRP3 inflammasome components, and TF, and constitute thrombo-inflammatory vectors that can be detected in the plasma from active JIA patients.

## 1 Introduction

IL-1β is a major cytokine in innate immunity, involved in the pathogenesis of various rheumatic diseases, notably systemic juvenile idiopathic arthritis (sJIA). Juvenile idiopathic arthritis (JIA) is a generic term to define pediatric inflammatory arthritis of unknown cause, which may occur with different clinical presentations. JIA could present as oligoarthritis, polyarthritis with or without circulating rheumatoid factor, psoriatic arthritis, enthesitis-related arthritis, and sJIA. sJIA is a rare form of the disease (10% of JIA) with systemic symptoms such as arthralgia, fever, evanescent rash, and a major biological inflammatory syndrome with neutrophilic polynuclear leukocytosis, elevated C-reactive protein (CRP), and hyperferritinemia, reflecting hypercytokinemia IL-1; notably, it has been shown to play an important role in the pathogenesis of sJIA and at a lower degree in other forms of JIA, since blocking IL-1 using the recombinant form of the IL-1 receptor antagonist or anti-IL-1β monoclonal antibody is an efficient strategy (1, 2). However, circulating IL-1β is usually not detectable, in patients, using immunoassays.

IL-1β belongs to the IL-1 family of cytokines, which, except for the IL-1 receptor antagonist, lack a leader sequence and do not follow the classical Golgi apparatus secretory pathway. This may in part, explain the low detectable circulating concentrations in the serum from patients (3). After priming through TLR activation and gene transcription, IL-1β is synthesized as a 33-kDa precursor. After a second activation cell signal, such as P2X7 purinergic receptor activation by ATP, an intra-cytoplasmic protein complex called inflammasome is assembled by the association of a NOD-like-receptor (NLR), mainly NLRP3, an adaptor-protein (ASC) and pro-caspase-1 (4). NLRP3 assembly and activation lead to pro-caspase-1 auto-cleavage in active caspase-1, followed by the processing of the IL-1β precursor in its 17-kDa mature form. The same mechanism is involved in the processing of IL-18, another important IL-1 family member (5). Following or concomitantly with this step, several mechanisms of IL-1β secretion have been proposed (6, 7). One may involve secretory lysosomes, which may be similar to autophagosomes and are released by cells when cytoplasmic autophagy is inhibited (8–11). Another mechanism may involve secretion of HLA class II-positive exosomes (12). When cells are more strongly activated or for a longer period of time, caspase-1 may induce IL-1β processing and the cleavage of gasdermin-D, which, in turn, induces a new type of cell death called pyroptosis (13–16). In this case, the 17-kDa mature IL-1β is released out of the cells through membrane pores (17). In addition, IL-1β may be released via another kind cell death independent of caspase-1, called necroptosis (18).

A different way of IL-1β release, barely detectable by usual assays, may be due to the shedding of membrane microvesicles (MVs) (19–21). MVs are small membrane extracellular vesicles (0.1–1 µm) shed by various cells during activation or cell death (22). Their role as a vector of bioactive molecules has been suggested, since MVs bear cell-surface receptors, contain various cytosolic proteins including cytokines, signaling molecules, or RNA, depending on cell origin and the kind of stimulus that induces their formation (23). Exposure of phosphatidylserine (PS) resulting from loss of plasma membrane symmetry is essential for the formation of MVs and enables their detection by flow cytometry, using annexin-V labeling (24). MVs are thought to be involved in many processes such as coagulation, inflammation, and angiogenesis and have been identified as potential biomarkers of diseases with high vascular risk (25). Many cellular types can release MVs, among them myeloid cells (26). MVs issued from activated monocytes or macrophages have been shown to contain bioactive IL-1β *in vitro*; however, their pro-inflammatory functions *in vivo* as well as their detection in human diseases have not been reported to date. Here, we sought to evaluate IL-1β-positive MV pro-inflammatory functions, using *in vitro* and *in vivo* models and tried to detect them in patients with JIA, in a pilot study.

## 2 Materials and methods

### 2.1 Cell culture

The cells used were macrophages differentiated from THP-1 cells treated with phorbol myristate acetate (PMA, Sigma-Aldrich, USA) for 24 h or from peripheral blood mononuclear cells (PBMCs) treated with GM-CSF (Sargramostim, 50 ng/mL) for 7 days and human umbilical vein endothelial cells (HUVECs). All culture conditions are described in **Supplementary Methods**.

### 2.2 Generation, numeration, and characterization of MV population

Cell culture media issued from the different conditions of stimulation were collected and centrifuged at 300*g* for 10 min, to eliminate cellular debris. Supernatants were centrifuged at 4,500*g* for 10 min at 4°C to remove apoptotic bodies, then at 70,000*g* for 90 min to pellet MVs. MVs were washed with phosphate buffered saline (PBS), resuspended in PBS, and stored at −80°C until being used. MVs were analyzed using a Gallios flow cytometer (Beckman Coulter, USA). Protocol settings and gates as forward scatter by side scatter were adjusted by using 0.5-, 0.9-, and 3-µm Megamix beads (Biocytex, France), as previously described (27). Isolated MVs were stained with 250 µg/mL FITC-labeled annexin A5 Kit (Tau Technologies, the Netherlands) for 30 min at room temperature in the dark, before adding 500 µL of annexin-V-binding buffer and flow-counting beads (Biocytex, France) (28).

To detect the presence of IL-1β, MVs were permeabilized in PBS containing 0.2% saponin, which, at this low concentration, guaranteed preservation of the integrity of the MV phospholipid bilayer, and then incubated at 4°C for 1 h with 25 µg/mL Alexa Fluor 647 anti-human IL-1β antibody (BioLegend, clone JK-1B-1, Cat# 508207, RRID: AB_604133, USA) or 25 µg/mL Alexa Fluor 647 mouse IgG1 control isotype (BioLegend Cat# 400155, RRID: AB_2832978). After washing, permeabilized MVs were stained with annexin-V-FITC to separate MVs from background in flow cytometry analysis. The same permeabilization procedure was used for double labeling of IL-1β and P2X7R, with the further use of an anti-P2X7R rabbit monoclonal antibody (Cell Signaling Technology Cat# 13809, RRID: AB_2798319) or a control rabbit IgG (Cell Signaling Technology Cat# 2729, RRID: AB_1031062), followed by addition of Alexa 488-conjugated goat anti-rabbit IgG (Thermo Fisher Scientific Cat# A78953, RRID: AB_2925776).

### 2.3 Western blot experiments

Cells and MVs were lysed in buffer containing 20 mM tris, pH 7.5,150 µM NaCl, 2 mM EDTA pH 8, 0.1% NP40, and a cocktail of protease inhibitors (Thermo Fisher Scientific, USA). Protein concentrations were measured using the BCA protein assay kit (Thermo Fisher Scientific, USA). For Western blot analysis, equal amounts of proteins (15 to 30 µg) were separated on a 4%–12% gradient Novex gel (Thermo Fisher Scientific, Waltham, United States), and then transferred on nitrocellulose membranes (GE Healthcare, USA). Blots were blocked with TBST (150 mM NaCl, 50 mM Tris-HCl at pH 7.5, and 0.1% Tween 20) containing 5% BSA for 1 h, then probed with the following primary antibodies: mouse anti-human IL-1β 1:1,000 (3A6, Cell Signaling Technology Cat# 12242, RRID : AB_2715503), rabbit anti-caspase-1 p10 1:200 (Santa Cruz Biotechnology Cat# sc-515, RRID : AB_630975), rabbit anti-human caspase-1 1:200 (Santa Cruz Biotechnology Cat# sc-622, RRID : AB_2069053), mouse anti-human NLRP3 1:1,000 (Enzo Life Sciences Cat# ALX-804-819-C100, RRID : AB_2051972), rabbit anti-human P2X7 1:200 (Sigma-Aldrich Cat# P9122, RRID : AB_477418), rabbit anti-human tissue factor (TF) 1:1,000 (Abcam Cat# ab151748, RRID : AB_2814773), rabbit anti-human ICAM-1 1:1,000 (Cell Signaling Technology Cat# 4915, RRID : AB_2280018), rabbit anti-E-selectin 1:200 (Santa Cruz Biotechnology Cat# sc-14011, RRID : AB_2186684), and rabbit anti-human VCAM-1 1:200 (Santa Cruz Biotechnology Cat# sc-8304, RRID : AB_2214058). Membranes were then incubated with horseradish peroxidase-conjugated secondary antibodies (Thermo Fisher Scientific, USA), using ECL or ECL Plus substrates (Thermo Fisher Scientific, USA) and chemiluminescence detection system (G:Box, Synoptics, UK).

### 2.4 Cytokine assays

After the lysis of MVs by three freeze–thawing cycles, IL-1β concentrations were measured using a highly sensitive ELISA assay (IL-1 beta Human ELISA Kit, High Sensitivity, Life Technologies, Cat # BMS224HS USA). The same ELISA kit was used for measuring IL-1β concentration in blood samples.

### 2.5 Tissue factor activity

TF activity was measured using a Factor Xa generation assay, as previously described (29). MVs were resuspended in HEPES (Sigma, St Louis, United States), and then incubated in a 96-well plate with anti-TF monoclonal antibody (clone 183 SBTF-1, BioCytex, Marseille, France) or control isotype (clone a-DNP 2H11–2H12, BioCytex, Marseille, France) antibodies for 30 min. A reaction mix containing FVII, Factor X, and CaCl_2_ (Sigma, St Louis, United States) was then added to each well to trigger the coagulation cascade and generate FXa. After 2 h of incubation, FXa activity was monitored by the addition of a fluorogenic substrate (factor Xa substrate, Stago 390/450nm, Biocytex, Marseille) (30) and fluorescence was measured, using a fluorimeter (Fluoroskan, Thermo Scientific, United States).

### 2.6 Transmission electronic microscopy

After thawing, MVs were centrifuged at 70,000*g* for 90 min at 4°C, then fixed in a PBS solution containing 2% paraformaldehyde and 0.2% glutaraldehyde. A 3-µL aliquot was applied on 100-mesh nickel formvar-coated grids (Euromedex, France). After 15 min, MVs were permeabilized with 3 µL of PBS–5% BSA–0.1% saponin for 1 h, then incubated for 1 h with anti-TF rabbit monoclonal antibody (Sigma-Aldrich clone 2L10 ZooMAb^®^, Cat# ZRB1811, RRID : AB_2938655) and anti-IL-1β mouse antibody (Cell Signaling Technology Cat# 12242, RRID : AB_2715503) diluted 1:25 in PBS–0.3% BSA. After three washes in PBS, MVs were labeled for 1 h with goat anti-rabbit IgG coupled with 10-nm gold beads (Thermo Fisher Scientific Cat# N-24916, RRID : AB_2539796) and goat anti-mouse IgG coupled with 5-nm gold beads (Tebu-Bio, Cat# 220GA1003, RRID : AB_2938656). Immunogold-labeled MVs were fixed for 5 min in cacodylate buffer containing 2.5% glutaraldehyde, then grids were negatively stained with 0.3% phosphotungstic acid for 1 min, before observation with a transmission electron microscope (JEM 1400 JEOL, United States).

### 2.7 Endothelial cell activation by MVs

MVs isolated from macrophages were incubated with HUVECs for 4 h at 37°C. Cell pellets were labeled for 1 h with specific anti-ICAM-1/CD54-PE (Beckman Coulter Cat# IM1239U, RRID : AB_131186), anti-VCAM-1/CD106-PE (Beckman, A66085, RRID : AB_2938658), anti-E-selectin/CD62E-PE (BioLegend, HCD62E RRID : AB_2938659), or control isotype antibodies IgG1-PE (Beckman Coulter Cat# A07796, RRID : AB_2832963) and IgG2aκ-PE (BioLegend Cat# 400213, RRID : AB_2800438) (5 µL/reaction) for flow cytometry analysis. For Western blot experiments, cells were washed and scraped in PBS, centrifuged, and lysed in RIPA buffer.

### 2.8 Animal studies

Mice experiments were conducted according to the institutional ethical committees for animal care from the local animal ethics committee of Marseille. C57BL/6J mice received intraperitoneal injections of PBS or IL-1 receptor antagonist IL-1Ra (30 mg/kg), with or without subsequent one peritoneal injection of 25×10^6^ MVs in PBS. Peripheral blood and peritoneal fluids were collected. The details of experimental conditions are described in **Supplementary Methods**.

### 2.9 Patient cohort

Ten patients with JIA according to the International League of Associations for Rheumatology (ILAR) definition were recruited from the Department of Paediatrics at Marseille and Nimes hospitals, during 2015–2016. Parents (or patient’s legal guardians) and patients received information and consent of parents (or patient’s legal guardians) was obtained for all individual participants included in the study. Clinical presentations consisted mainly in refractory polyarticular and systemic forms of the disease, but all patients presented active disease defined as AJI flare according to Juvenile Arthritis Disease Activity Score 10 (JADAS10). The main demographic, clinical, and biological characteristics are shown in **Table 1**. Ten healthy adult donors were also included.

**Table 1:** Characteristics of patients with active juvenile idiopathic arthritis (JIA).

Patients	Age	Sex	Form	Time of Evolution	ANA	HLAB27	Clinical A.	Arthritis	Ocular Manifestation	Systemic A.	Biological A.	Current Treatment	Previous Treatment
1	16	F	polyA	16	–	neg	yes	hips knees	uveitis	no	CRP = 3 mg/L ESR = 13 mm HG normal	Abatacept + MTX	CS DMARDS Etanercept Adalimumab
2	9	F	polyA	3	–	neg	yes	hips knees ankles	no	no	CRP = 2 mg/L ESR = 3 mm HG normal	Tocilizumab	CS DMARDS Etanercept Adalimumab Abatacept Tocilizumab
3	13	M	oligoA	11	–	neg	yes	hips	no	fever rash	CRP = 3 mg/L ESR = 20 mm HG normal	Tocilizumab	CS DMARDS Etanercept Anakinra
4	10	M	polyA	2	–	neg	yes	cuffs hips	no	fever rash	CRP = 74 mg/L ESR = 30 mm PNN: 12,000/mm^3^	CS	CS
5	16	F	polyA	5	RF + ANA + CCP +	neg	yes	ankles cuffs fingers	no	no	CRP = 2 mg/L ESR = 5 mm HG normal	Abatacept + MTX	CS DMARDS Adalimumab Etanercept Adalimumab + MTX Abatacept + MTX Tocilizumab
6	7	F	polyA	6	–	neg	yes	knees cuffs hips	uveitis	no	CRP = 3 mg/L ESR = 8 mm HG normal	Tocilizumab + MTX	CS DMARDS Etanercept Adalimumab Abatacept
7	16	F	polyA	14	–	neg	yes	elbows cuffs knees ankles fingers	no	fever rash	CRP = 3 mg/L ESR = 30 mm HG normal	Abatacept + MTX	CS DMARDS Etanercept + MTX Adalimumab + MTX Abatacept
8	5	M	polyA	3	–	neg	yes	knees cuffs hips	no	fever rash	CRP = 9 mg/L ESR = 2 mm HG normal	Tocilizumab	CS Anakinra Tocilizumab
9	8	M	polyA	1	–	neg	yes	fingers elbows knees cuffs	no	fever	CRP = 230 mg/L ESR = 50 mm Ferritin = 883 µg/L PNN: 23,000/mm^3^	MTX	CS Anakinra MTX
10	11	M	polyA	5	–	neg	yes	hips shoulders	no	fever	CRP = 122mg/L ESR = 45 mm Ferritin = 309 µg/L PNN: 17,400/mm^3^	–	CS MTX Tocilizumab

Demographic characteristics, clinical and biological disease activities, and treatments for 10 patients with active JIA are described.

Age, years; F, female; M, male; PolyA, polyarticular; OligoA, oligoarticular; ANA, anti-nuclear antibodies; RF, rheumatoid factor; CCP, anti cyclic-citrullinated peptide antibody; neg, negative; Clinical A, clinical activity; Systemic A, systemic activity; Biological A, biological activity; CRP, C-reactive protein; ESR, erythrocyte sedimentation rate; HG, hemogram; PNN, polynuclear neutrophils; CS, corticosteroids; DMARDS, disease-modifying antirheumatic drugs; MTX, methotrexate.

### 2.10 Patient blood sampling and platelet-poor plasma preparation

Sampling was justified by the flare of the disease. A fasting blood sample was obtained by venipuncture in citrate-containing tubes, which were centrifuged twice at 2,500*g* for 15 min at room temperature in order to obtain platelet-poor plasma (PPP), within a maximum of 2 h after blood collection, as previously described (31). The resulting plasma was divided into aliquots and stored at −80°C until analysis.

### 2.11 Determination of patients’ MV profile by flow cytometry

PPP aliquots were thawed at room temperature and centrifuged at 20,000*g* for 90 min to pellet MVs. Resuspended MVs (30 µL) were transferred into different tubes for MV labeling. Annexin V-FITC (Tau Technologies, the Netherlands) and conjugated antibodies (Ab) were added to identify specific MV subsets: CD31-Phycoerythrin (PE) Ab (Beckman Coulter Cat# IM2409, RRID : AB_131205), CD41-PC7 Ab (Beckman Coulter Cat# 6607115, RRID : AB_2800448), CD11b-APC Ab (Beckman Coulter Cat#A87782, RRID : AB_2938661), CD235a-Alexa 750 Ab (Beckman Coulter Cat# A89314, RRID : AB_2938662), CD14-APC Ab (Beckman Coulter Cat# IM2580, RRID : AB_2800451), CD66b-APC Ab (Beckman Coulter Cat# B08756, RRID : AB_2893284), and CD11b-PE Ab (Beckman Coulter Cat# IM2581U, RRID : AB_131334). The numeration of total MVs and MVs derived from specific cell subsets (absolute number/mL plasma) were calculated according to the acquired counts of MVs and microbeads and the dilution performed during sample preparation.

### 2.12 Statistical analysis

Data represent the cumulative results of all experiments and are expressed as median with interquartile range for the indicated number (*n*) of experiments. Statistical analysis was performed with Prism version 9 software. Significant differences were determined by using nonparametric Mann–Whitney and Wilcoxon test; *p*-values < 0.05 were considered statistically significant.

## 3 Results

### 3.1 MVs from stimulated macrophages contain NLRP3 inflammasome components necessary for caspase-1-dependent IL-1β processing

**Figure 1:**
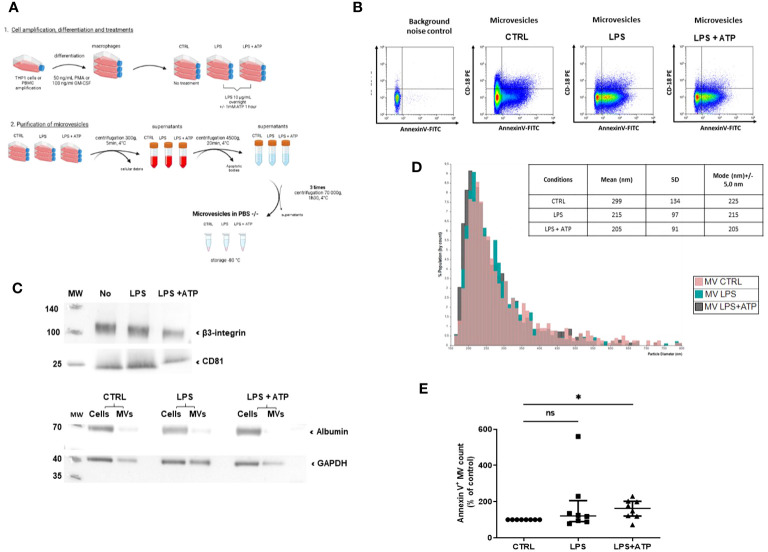
Macrophage MV purification and characterization process. **(A)** Schematization of the preparation and the purification of MVs. MVs were collected from macrophage supernatants (purified THP-1 treated with PMA), either non-stimulated (CTRL) or stimulated with LPS or LPS+ATP. Then, they were purified by serial centrifugations to remove dead cells and apoptotic bodies and ultracentrifugation. **(B)** Flow-cytometry-based quantification of MVs collected from non-stimulated or LPS- or LPS+ATP-stimulated macrophage supernatants. MVs were characterized by annexin-V and CD18 expression. **(C)** After lysis, 17, 150, or 236 millions of MVs (CTRL, LPS, and LPS+ATP) were analyzed by Western blot to detect the presence of β3-integrin and CD81 (upper panel). The same number of MVs and 10 µg of their corresponding cells were lysed and a Western blot of albumin was realized to attest purification. GAPDH was used as charge control (lower panel). **(D)** Size distribution by tunable resistive pulse sensing (qNANO) with p150 nanopores. **(E)** MVs isolated from THP-1 treated with PMA and differentiated into macrophages and non-stimulated (CTRL) or stimulated by LPS or LPS+ATP were stained with annexin-V-FITC, and then quantified by flow cytometry, using counting beads. Results are expressed as mean percentage of increase compared to control expressed in each experiment at 100% (ns, *p* > 0.05; *p < 0.05, *n* = 8).

According to the Minimal Information for Studies of Extracellular Vesicles (MISEV) 2018 guidelines (32), MVs issued from THP-1 differentiated into macrophages by PMA treatment were then purified by successive centrifugations and ultracentrifugations (**Figure 1A**). MVs from unstimulated and stimulated macrophages were characterized using several methods. First, using flow cytometry, the classical gating strategy was determined (**Supplementary Figure S1**). Then, the presence of typical membranous and cytosolic MV-associated molecules was confirmed by detecting CD18 (integrin subunit β2) and Annexin-V binding on purified MVs (**Figure 1B**). In addition, the presence of integrin subunit β3 and CD81 was confirmed by Western blotting (**Figure 1C**, upper panel), with no major contamination by soluble proteins such as albumin (**Figure 1C**, lower panel). Then, tunable resistive pulse sensing assays showed that vesicles had similar average sizes (respectively 299, 215, and 205 nm for conditions CTRL, LPS, and LPS+ATP), which correspond to the range of large MVs (**Figure 1D**). Taken together, molecular and size features of MV preparations were consistent with the definition of MV according to the MISEV 2018.

To determine the biological activity of IL-1β conveyed by MVs, we used MVs purified from supernatants of THP-1 cells differentiated into macrophages by PMA treatment and stimulated with LPS or LPS+ATP. MV numeration showed that the same number of MVs were issued from LPS-stimulated or non-stimulated macrophages (ns, **Figure 1E**). Further addition of ATP tended to increase the total number of MVs compared to non-stimulated macrophages, despite heterogeneous results (58% increase, *p* < 0.05, **Figure 1E**). Similar results were obtained using MVs from PBMC differentiated into macrophages (*p* < 0.005, **Supplementary Figure S2**).

**Figure 2:**
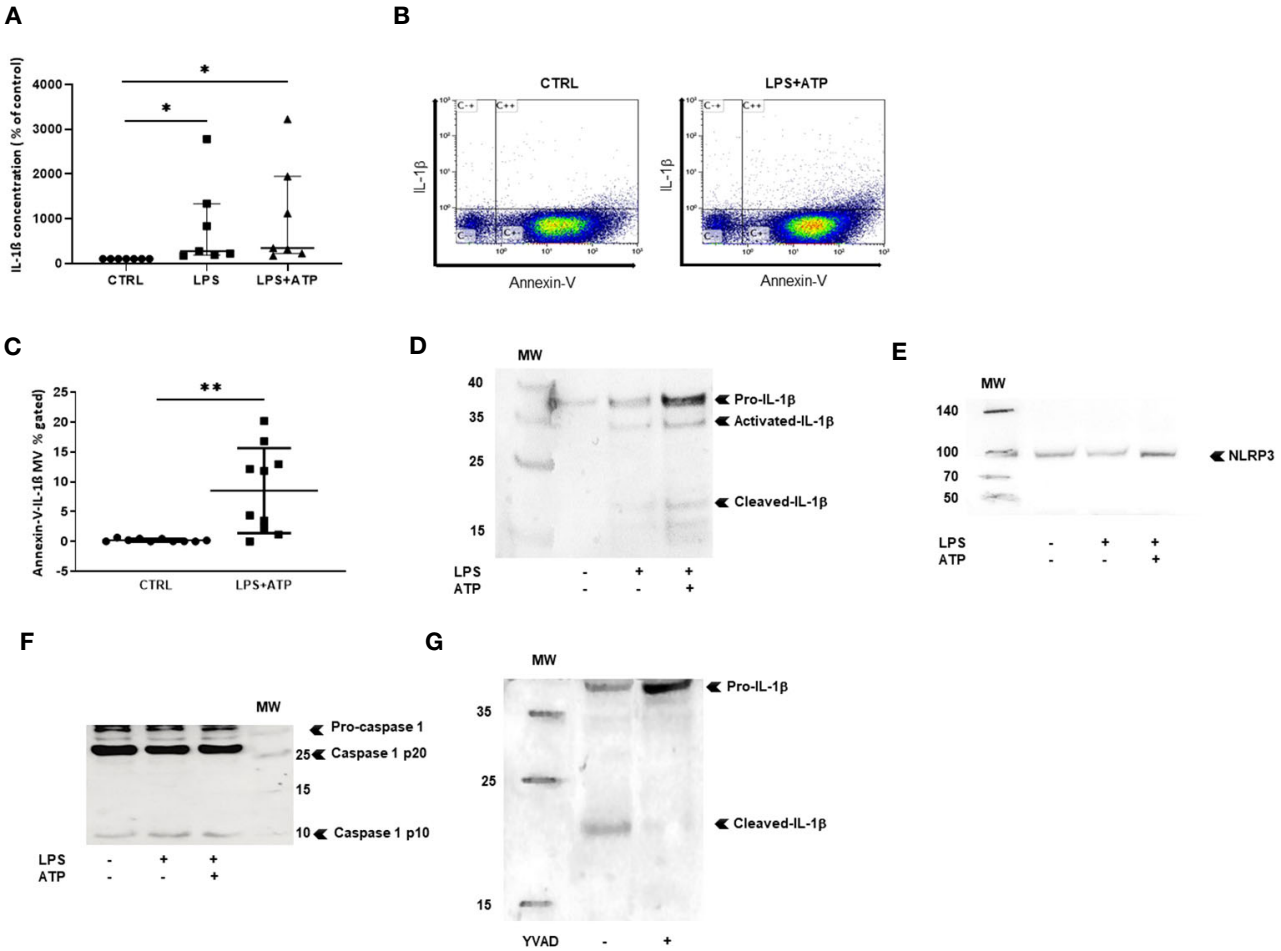
MVs shed from stimulated macrophages convey IL-1b and NLRP3 inflammasome components. **(A)** IL-1b was measured by ELISA in MV lysates isolatedfrom PBMC differentiated into macrophages stimulated with either LPS or LPS+ATP or non-stimulated (CTRL). Results are expressed as percentageof increase compared to control (*p < 0.05, n = 7). **(B)** Scatter dot plot after MV purification, permeabilization, and co-labeling with annexin-V andIL-1b. **(C)** MVs were isolated from THP-1 differentiated into macrophages. Quantification of annexin-V and IL-1b-positive MVs (**p < 0.01, n = 10).Detection of **(D)** IL-1b, **(E)** NLRP3, and **(F)** pro-caspase-1 and mature caspase-1 (p10) and (p20) in MV lysates from THP-1 differentiated intomacrophages, by Western blotting, **(G)** detection of IL-1b by Western blotting in MV lysates from THP-1 differentiated into macrophages aftertreatment with the caspase-1 inhibitor YVAD. All Western blots were realized at least three times.

We then studied NLRP3 inflammasome components carried by MVs from THP-1 treated with PMA and differentiated in macrophages. Using ELISA, we observed that IL-1β concentration was significantly higher in MV lysates issued from LPS- and LPS+ATP-stimulated macrophages compared to MV lysates from non-stimulated macrophages (*p* < 0.05, **Figure 2A**). We used flow cytometry to detect IL-1β in MVs after membrane permeabilization by saponin, as previously reported (33) (**Figure 2B**) and observed a significant increase of IL-1β-positive MV population shed from macrophages stimulated with LPS+ATP as compared to non-stimulated ones [8.1 (2–13.9) vs. 0.115 (0.03–0.475)%, *p* < 0.01, **Figure 2C**].

Western blot experiments were performed only with MVs purified from the supernatant of PMA-treated THP-1 cells, as large amounts of MVs were required for protein expression analysis. MVs from non-stimulated macrophages contained small concentrations of the IL-1β precursor (33 kDa), likely resulting from THP-1 cell activation by PMA. After LPS stimulation, increased levels of IL-1β precursor (pro-IL-1β) and very low amounts of mature IL-1β (17 kDa) as well as intermediary IL-1β (28 kDa) form were observed, suggesting that priming with LPS induced pro-IL-1β synthesis by macrophages. With the addition of exogenous ATP, IL-1β mature form increased in MVs, consistent with ATP-induced pro-IL-1β maturation (**Figure 2D**).

We then investigated the presence of NLRP3 and caspase-1 in MVs from THP-1 treated with PMA, differentiated in macrophages. Using Western blot, NLRP3 (**Figure 2E**) and the 40-kDa procaspase-1 and the 10-kDa mature and 20-kDa mature caspase-1 forms (**Figure 2F**) were clearly detected in MVs secreted by stimulated macrophages, indicating that MVs issued from activated macrophages contained all the machinery necessary for the processing of IL-1β. In addition, when PMA-treated THP-1 cells were pre-treated by caspase-1-inhibitor YVAD then stimulated with LPS+ATP, MV content was modified showing less IL-1β mature form and increased concentrations of pro-IL-1β (**Figure 2G**), demonstrating that IL-1β maturation in MVs was caspase-1-dependent.

### 3.2 MVs from stimulated macrophages are thrombo-inflammatory vectors

**Figure 3:**
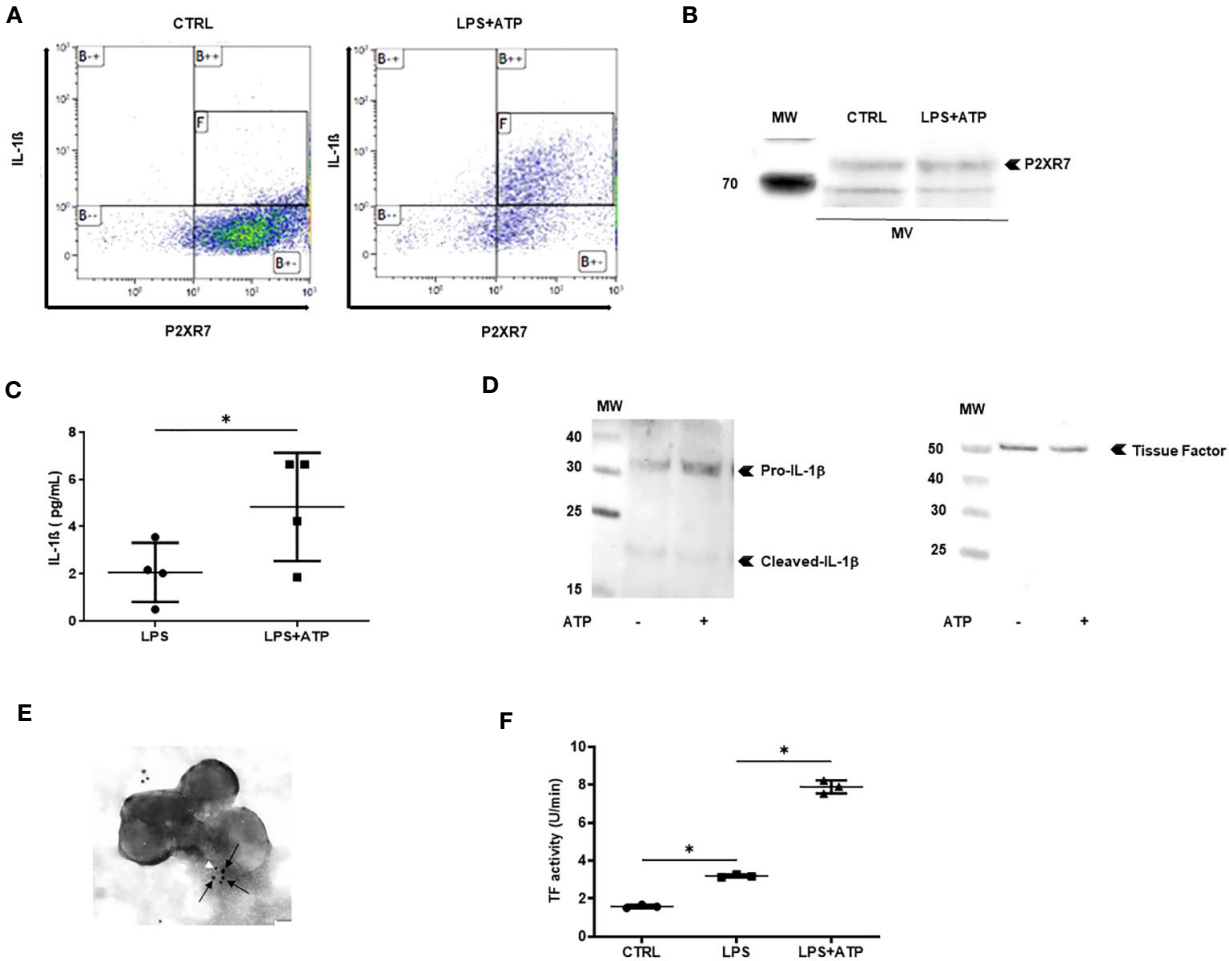
MVs from stimulated macrophages are thrombo-inflammatory vectors. **(A)** Detection by flow cytometry of IL-1β and P2X7R in permeabilized annexin-V-positive MVs isolated from PBMC differentiated into macrophages then stimulated with LPS+ATP or not stimulated (CTRL). Quadrant [F] shows MVs positive for IL-1β and P2X7R among annexin-V-positive MVs. **(B)** P2X7 was detected by Western blot in MVs or cell lysates from PBMC differentiated into macrophages then stimulated with LPS+ATP or not stimulated. **(C)** MVs purified from LPS-stimulated THP-1 differentiated into macrophages were incubated with PBS or ATP (1 mM) for 1 h at 37°C. IL-1β concentration was determined in MV supernatant using ELISA (*p < 0.05, *n* = 4). **(D)** Detection of IL-1β (left) and tissue factor (right) by Western blot in 10 µg of MV lysate purified from THP-1 treated with PMA and differentiated into macrophages, then stimulated with LPS+ATP. **(E)** Immunogold labeling of IL-1β (white arrowhead, 5 nm beads) and tissue factor (black arrows, 10 nm beads) captured by transmission electronic microscopy in MVs from THP-1-derived macrophages stimulated with LPS+ATP. **(F)** Tissue factor activity (U/min) measured using a Factor Xa generation assay in MVs from THP-1-derived macrophages incubated with PBS (CTRL) or stimulated with either LPS or LPS+ATP (*p < 0.05, *n* = 3).

Next, we studied the pro-inflammatory and pro-coagulant properties of IL-1β-positive MVs. Since MVs contain all the molecules necessary to generate the 17-kDa mature IL-1β, we asked whether they could process and release IL-1β *in vitro.* First, using flow cytometry analysis and gating on large permeabilized Annexin-V-positive MVs, in order to reduce exosome contamination, we observed co-expression of IL-1β and P2X7 receptor in and on the same MV. More than 80% of MVs issued from non-stimulated macrophages expressed only P2X7R, whereas permeabilized MVs issued from LPS+ATP-stimulated macrophages expressed both P2X7R and IL-1β (**Figure 3A**). Western blot analysis confirmed the presence of P2X7R in MV lysates (**Figure 3B**). MVs were purified from LPS-stimulated macrophages before being incubated with PBS or ATP. After ultracentrifugation to eliminate MVs, IL-1β concentration was measured in MV-free supernatant and a threefold increase of IL-1β concentrations was observed in the supernatant of MVs stimulated with ATP, compared to the supernatant of MVs stimulated with PBS alone (*p* < 0.05, **Figure 3C**).

MVs issued from macrophages have been reported to carry TF (34); we thus asked whether MVs contained both IL-1β and TF. MVs were isolated from supernatants of macrophages derived from THP-1 and MV lysates were subjected to Western blot analysis. Both IL-1β and TF could be detected in the same MV issued from LPS+ATP-stimulated macrophages (**Figure 3D** left and right panels, respectively). Moreover, using transmission electronic microscopy (TEM) on MV double-labeled for both IL-1β and TF, we observed that some MVs contained both IL-1β and TF (**Figure 3E**). To confirm TF procoagulant activity on MVs, an *in vitro* Factor-Xa-generation-assay was performed. TF activity was significantly increased with MVs issued from LPS+ATP-stimulated macrophages, when compared with MVs issued from LPS-stimulated macrophages and non-stimulated macrophages (*p* < 0.05 respectively, **Figure 3F**).

### 3.3 MVs from stimulated macrophages have pro-inflammatory effects *in vitro* and *in vivo*

**Figure 4:**
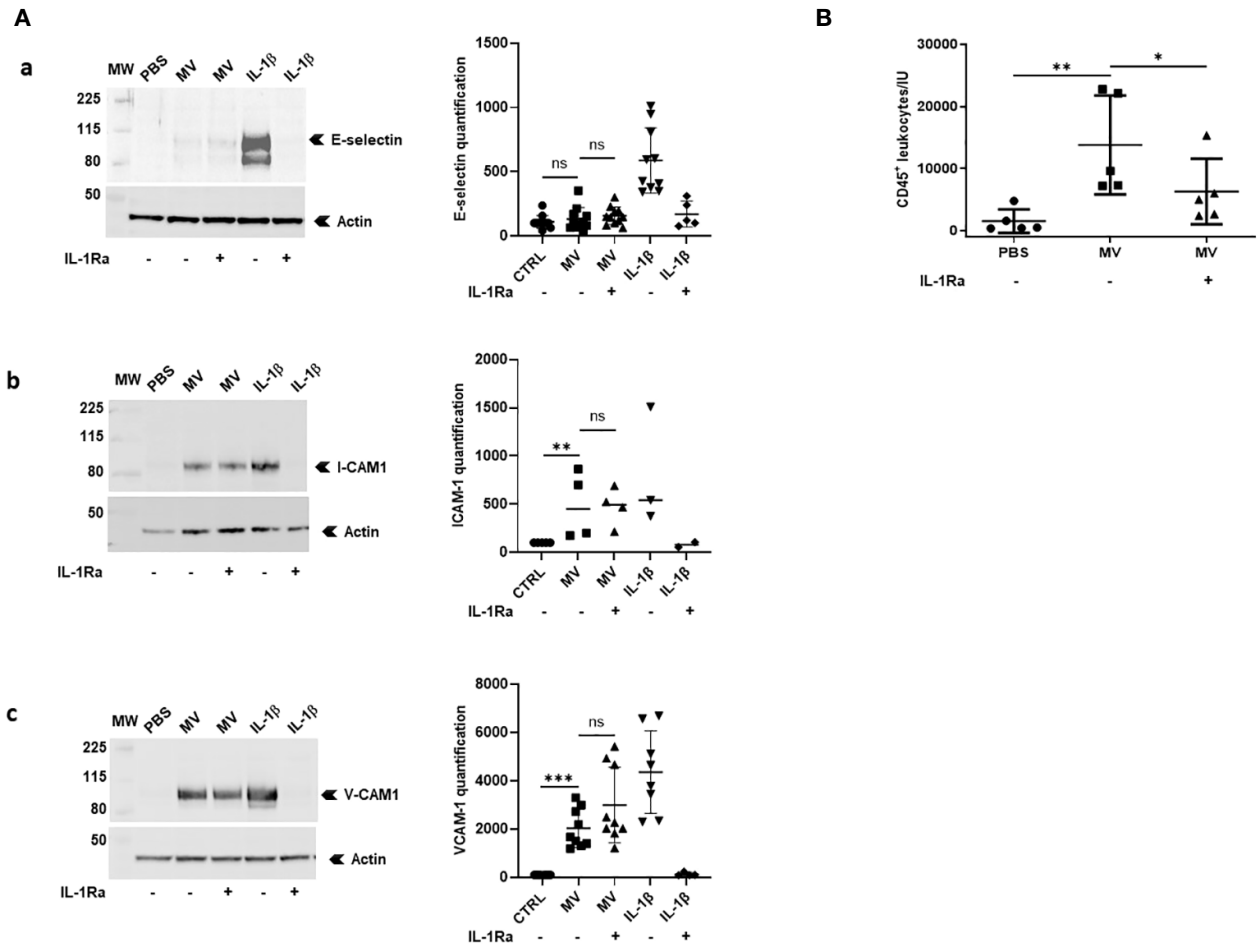
MVs from stimulated macrophages demonstrate IL-1β-dependent pro-inflammatory properties *in vitro* and *in vivo.* **(A)** HUVECs were pre-incubated or not with IL-1Ra (10 µg/mL) for 10 min and were then incubated with PBS or with 20 × 10^6^ MVs from THP-1-derived macrophages stimulated with LPS+ATP or with IL-1β (500 pg/mL). Cell lysates (30 µg) were then subjected to Western blot for ICAM-1, VCAM-1, E-selectin detection, and actin for each point (for MV stimulations: ICAM-1: *n* = 4, VCAM-1: *n* = 9, and E-selectin: *n* = 11; ns, *p* > 0.05, **p < 0.01, ***p < 0.001). **(B)** Peritoneal leukocyte counts after peritoneal injection in mice (*n* = 5) of 25×10^6^ MVs from THP-1-derived macrophages treated with LPS+ATP. Four mice received intraperitoneal injections of PBS or IL-1Ra (30 mg/kg) 30 min before the MV injections (*p < 0.05, **p < 0.01).

To determine whether IL-1β-positive MVs were biologically active, we first studied the ability of MVs issued from LPS+ATP-stimulated macrophages to activate endothelial cells *in vitro*. Using Western blotting, we observed that MVs significantly increased ICAM-1 and VCAM-1 expression (*p* < 0.01 and *p* < 0.001, respectively, **Figure 4A**, upper panel a and middle panel b) but not E-selectin expression on HUVECs (ns, **Figure 4A**, lower panel c). Interestingly, MV pre-incubation with IL-1Ra did not reduce either ICAM-1 or VCAM-1 expression on endothelial cells (ns, **Figure 4A**). Endothelial cell activation was also confirmed by cytometry analysis on HUVECs using the same conditions (**Supplementary Figure S3**; **Supplementary Table 1**).

Then, we studied the ability of MVs issued from LPS+ATP-stimulated macrophages to induce leukocyte recruitment in mice. Fifteen hours after intra-peritoneal injection of MVs, leukocyte recruitment (CD45+ cells) into peritoneal cavity was significantly increased (*p* < 0.01, **Figure 4B**), a process that was significantly reduced in the presence of IL-1 receptor antagonist (*p* < 0.05, **Figure 4B**).

### 3.4 Detection of IL-1β-positive MVs in patients with juvenile idiopathic arthritis

**Figure 5:**
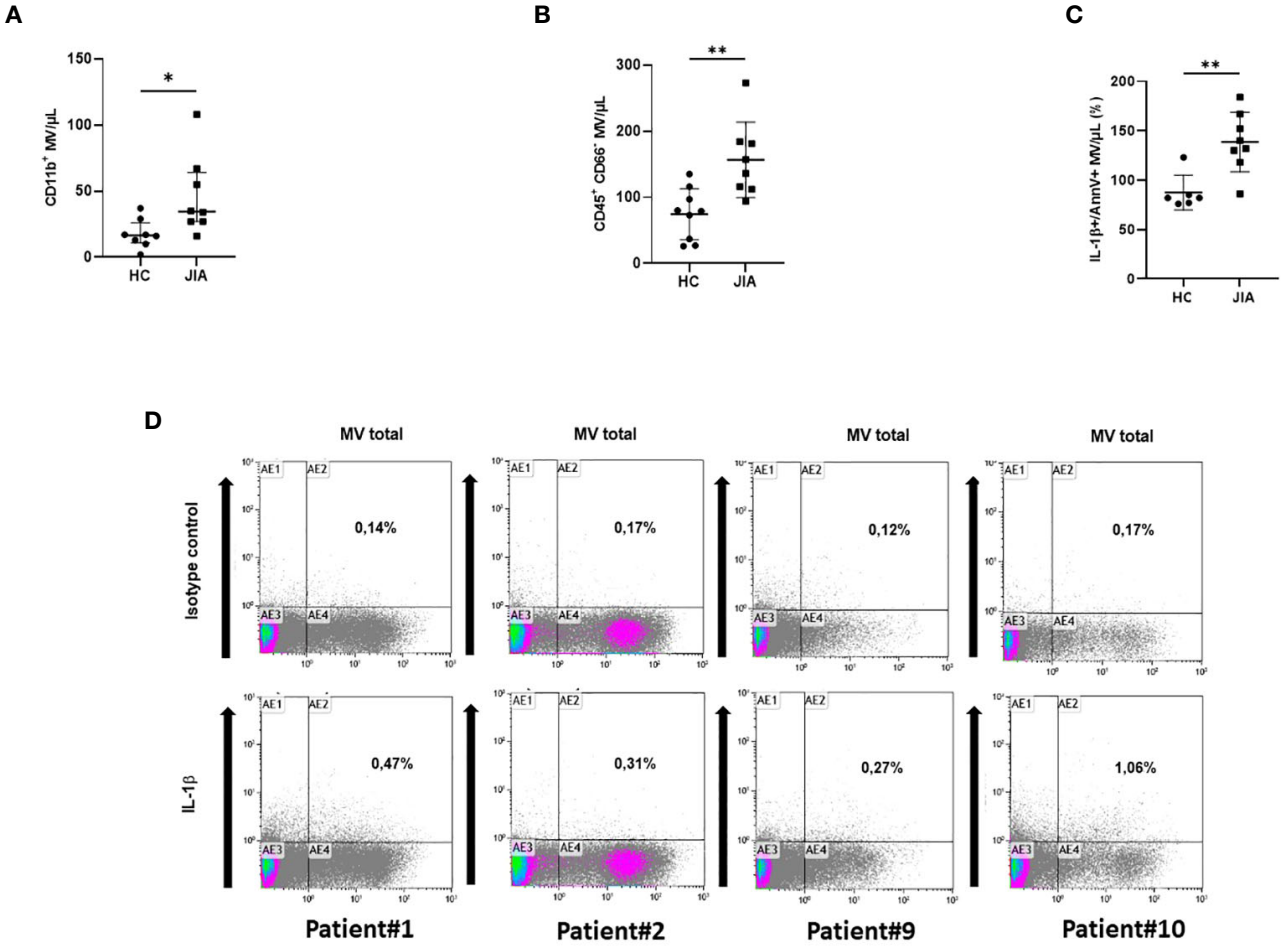
Detection of circulating IL-1β-positive MVs in plasma from JIA patients. **(A)** Determination by flow cytometry of myeloid MVs (CD11b+MV) isolated from the plasma of 10 JIA patients and 8 healthy controls (HC) and **(B)** of myeloid MVs after exclusion of neutrophils (CD45+CD66-MV). (*p< 0.05, **p< 0.01). **(C)** Detection by flow cytometry of circulating IL-1β-positive MVs among total MVs in 10 JIA patients versus six healthy controls (HC), expressed as percentage of the total annexin-V-positive MV population. **(D)** Illustration of dot plot from selected patients with active disease: #1, #2, #9, and #10. Percentage of IL-1β and annexin-V-positive MVs is expressed on each graph.

To support the existence of these MVs in human disease, we studied the presence of circulating MVs in the plasma of patients with JIA or from healthy donors. Ten patients (sex ratio = 1) with a mean age of 11.1 years [5–16 years], were studied (**Table 1**). All patients had active disease according to JADAS10, which motivate a therapeutic increment. Among them, nine patients presented a polyarticular form including three with systemic clinical and biological symptoms consistent with a systemic form (sJIA: patients #4, #9, and #10). No patient was treated with anakinra at the time of the flare. The absolute number of total MVs and of leukocyte-derived MVs were not significantly different in plasma of JIA patients compared to healthy controls, possibly due to the relatively low number of patients (data not shown). However, if the concentration of CD11b^+^MVs derived from myeloid cells was not significantly higher in JIA patients compared to healthy controls (ns, **Figure 5A**), CD45^+^CD66^-^MVs issued from myeloid cells after exclusion of neutrophils were significantly increased in JIA patients (*p* < 0.05, **Figure 5B**). Importantly, using flow cytometry analysis, we could detect higher concentrations of circulating IL-1β+Annexin-V-positive MVs in JIA patients compared to healthy controls (*p* < 0.05, **Figure 5C**). Circulating IL-1β+Annexin-V-positive MVs represented a small proportion of total MVs (**Figure 5D**) but were detectable in all patients, especially in those with an active polyarticular disease (patient#1: 0.47%, patient#2: 0.31%) and with biological systemic inflammation (patient#9: 0.27%, and patient #10: 1.06% respectively).

## 4 Discussion

In this study, we observed that macrophages issued from either PBMCs or the THP-1 cell line release pro-inflammatory and pro-thrombotic MVs upon priming with LPS and activation with ATP. MVs contained the precursor and mature forms of IL-1β, NLRP3 inflammasome, pro-caspase-1, and activated capsase-1 and expressed P2X7R on their membranes. Upon ATP stimulation, MVs were able to release IL-1β in its mature form *in vitro* and increased ICAM-1 and VCAM-1 expression on HUVECs. In addition, MVs induced a peritoneal leukocyte infiltrate in mice that was reduced by IL-1Ra pre-treatment. After ATP stimulation, MVs also contained TF and exhibited an increased TF-dependent pro-coagulant activity *in vitro*. As a proof of concept of their existence and potential pathogenic roles in human pathogenesis, using flow cytometry analysis, we could detect IL-1β-positive MVs in the plasma of patients with active JIA, a well-known IL-1β-mediated disease.

As a leaderless protein, IL-1β secretion does not follow the classical secretory pathway (3). Several alternative releasing pathways have however been proposed, notably linked to inhibition of autophagy and secretory lysosome exocytosis (8–11). Secretory lysosomes have been shown to contain pro- and mature IL-1β, NLRP3, ASC, and caspase-1 (8). Other secretion mechanisms may occur after more prolonged macrophage activation through caspase-1-dependent pyroptosis or cathepsin B-dependent necroptosis. In these cases, free mature IL-1β is secreted (16, 18). Our results argue for the release of mature IL-1β by MVs issued from activated macrophages in a process associated with NLRP3 inflammasome and caspase-1 activation. Extracellular vesicles contain several different subpopulations such as, first, apoptotic bodies labeled by annexin-V, containing DNA, and histones with a size range between 1 and 3 µm; second, annexin-V-positive membrane MVs containing RNA and proteins, whose size is between 0.1 and 1 µm; and third, exosomes, which are annexin-V-negative but contain HLA class II with a small size between 50 and 80 nm (35). Apoptotic bodies have been reported to contain bioactive IL-1α (33) and some exosomes may contain IL-1β (12). In 2001, McKenzie et al. first reported the evidence of pro- and mature IL-1β-positive vesicles issued from LPS+ATP-stimulated THP-1; however, no centrifugation procedure or size criteria were clearly reported in this previous study (20). In 2007, Pizzirani et al. showed the presence of both forms of IL-1β, as well as caspase-1 in 0.1–1 µm cathepsin B-positive microvesicles issued from ATP-stimulated dendritic cells, but in this work, no specific labeling was used for purification; thus, this extracellular preparation was likely to contain both MV and secretory lysosomes (21). In 2011, Wang et al. reported the presence of IL-1β in annexin-V-positive MVs issued from LPS-activated THP-1, without the addition of ATP (19). MVs contained both forms of IL-1β, NLRP3, ASC, and caspase-1 and were shown to stimulate HUVECs *in vitro*, in an IL-1β-dependent manner. Our data confirm the pro-inflammatory nature of IL-1β-positive MVs and extend the study of Wang et al. in several ways. First, we could detect and characterize MV population and, among them, IL-1β-positive MVs by flow cytometry analysis, according to MISEV 2018 guidelines. Second, in agreement with another report (21), we observed that these MVs co-express IL-1β and P2X7R and are sensitive to ATP stimulation for the release of soluble 17 kDa mature IL-1β. Third, since IL-1β-positive MV-induced endothelial activation appeared partially IL-1-dependent *in vitro*, we showed for the first time that IL-1β-positive MVs exert an IL-1-mediated pro-inflammatory effect *in vivo* in a murine model of sterile peritonitis.

Therefore, these MVs may be considered as complete pro-inflammatory circulating entities protecting both IL-1β and NLRP3 inflammasome components from rapid degradation. The presence of MVs containing various pro-inflammatory molecules has now been well-established in various pathogenic models, notably ischemic (36). However, MVs as well as apoptotic bodies expose phosphatidyl-serine on their membranes, which is an important “eat-me signal” for macrophages leading to M2 anti-inflammatory phenotype differentiation (37), raising the question of the real pro-inflammatory properties of MVs *in vivo*. In fact, the role of MVs may well depend on the balance between the MVs produced during tissue injury and the phagocytosis capacities of macrophages. In physiological, macrophages may phagocytose MVs, preventing inflammation. During acute ischemic stress, necrotic cells have been shown to liberate very large concentrations of MVs (38), which may temporarily exceed macrophage phagocytosis. Alternatively, in patients prone to develop auto-immune diseases, such as systemic lupus erythematosus, the intrinsic phagocytic abilities of macrophages have been shown to be altered and circulating MVs have been reported (39, 40). Both situations may lead to increased circulating MVs and localized or systemic inflammation. MVs may deliver an IL-1β-mediated pro-inflammatory signal in several ways. First, as shown in other models, MVs may be endocytosed by target cells through phosphatidyl serine/phosphatidyl serine receptor interactions (41). Noteworthy is the fact that in this setting, NLRP3 may remain active into the cell cytoplasm (42). Through their P2X7 receptor, MVs may also interact with ATP liberated by necrotic cells, thus activating NLRP3 into the MV cytoplasm and MV IL-1β release, as suggested by our *in vitro* data. Third, MVs may experience secondary necrosis and the release of IL-1β precursor, NLRP3, caspase-1, and other enzymes, leading to some IL-1β processing in the target cell microenvironment and interaction with the IL-1R1 on target cells (43).

MVs may be able to deliver bioactive IL-1β in inflammatory sites (synovium, skin, bone marrow, and liver) distant from the original cell source. Interestingly, IL-1β-positive MVs may also be inflammatory through phagocytosis or membrane fusion by targeting fibroblast-like-synoviocytes or macrophages and direct intra-cytoplasmic delivery of both NLRP3 inflammasome components and pro-IL-1β, as previously suggested (42). In addition, Rothmeier et al. have shown that LPS+ATP-stimulated murine macrophages release MVs over 500 nm, which carry a bioactive form of TF, but do not contain IL-1β (34). Unlike data from these authors, we were able to detect both IL-1β and bioactive TF in MVs issued from LPS+ATP-stimulated macrophages using Western blot and *in vitro* bioassay. However, we could not show co-expression of IL-1β and TF on the same MV, using flow cytometry analysis, since TF was not detectable on MVs using this method, despite the use of different anti-TF antibodies. Therefore, we performed TEM on IL-1β and TF-double-labeled MVs and observed that a sub-population of MVs issued from LPS+ATP-stimulated macrophages indeed contained both IL-1β and TF. The relative differences with the previous report from Rothmeier et al. may possibly be due to the different experimental conditions, since we used lower concentrations of ATP (1 mM vs. 4 mM), possibly protecting macrophage MVs from pyroptosis and 17-kDa IL-1β release.

By stimulating HUVECs with IL-1β-positive MVs, we observed that endothelial activation was not inhibited by IL-1Ra, indicating that MVs may contain other pro-angiogenic and pro-inflammatory components, such as mitochondria for example (44), or other IL-1 family cytokines such as IL-18 or IL-33, which could induce endothelial activation in an IL-1β-independent manner (45). Membrane interaction of MVs could also be involved in endothelial activation. MVs expose phosphatidyl-serine on their membrane, resulting in an interaction with their endothelial phosphatidyl-serine receptor linked to ICAM-1 and VCAM-1 expression (46). *In vivo*, the pro-inflammatory properties induced by IL-1β-positive MVs injected into the peritoneal cavity could involve endothelial–leukocyte adhesion molecules (ICAM-1, VCAM-1) but also other mechanisms induced by IL-1β, such as regulation of chemokines, cytokine-induced neutrophil chemoattractant-1 (CINC-1), or integrin-β6 expression by epithelial cells (45). Thus, the permeability of the vasculature could be increased by regulating the expression of cell–cell junction components in an IL-1β-independent manner, and IL-1β can also induce the activation of leukocytes, fibroblast-like-synoviocytes, and endothelial cells, which could modify the vasculature permeability (47). Thus, MVs from stimulated macrophages are thrombo-inflammatory vectors and induce endothelial activation *in vitro* and leukocyte recruitment *in vivo*, in an IL-1β-dependant manner.

Finally, to determine whether our *in vitro* and murine data had some relevance in human diseases and as a proof of concept, we tried to detect IL-β-positive MVs in active JIA, a well-known IL-1β-mediated disease. In JIA patients, serum concentrations of IL-1β remain poorly detectable despite the demonstration of an IL-1β transcriptional signature in PBMCs and the well-established therapeutic efficiency of IL-1β-blocking antibody canakinumab or recombinant IL-1 receptor antagonist, anakinra (1, 48). We succeeded in detecting circulating IL-1β-positive MVs in patient plasma and observed a significantly higher count of myeloid IL-1β-positive MVs in 10 active JIA patients, compared to healthy controls. To our knowledge, this is the first time that IL-1β-positive MVs are detected in plasma from JIA patients.

## 5 Conclusion

In this study, we characterized a population of LPS-primed macrophage-derived MVs containing pro-IL-β, NLRP3, caspase-1, and TF and releasing IL-1β upon P2X7R activation by ATP. We showed their pro-inflammatory and pro-coagulant biological functions *in vitro* and *in vivo.* Furthermore, IL-1β-positive MVs were detectable in plasma from patients with active JIA; thus MVs may represent a pathway of IL-1β release in IL-1β-dependent diseases.

## Data availability statement

The original contributions presented in the study are included in the article/**Supplementary Material**. Further inquiries can be directed to the corresponding author.

## Ethics statement

Ethical approval was not required for the studies on humans in accordance with the local legislation and institutional requirements because only commercially available established cell lines were used. The animal studies were approved by APAFIS#4228-2016022410055380 v5. The studies were conducted in accordance with the local legislation and institutional requirements. Written informed consent was obtained from the minor(s)’ legal guardian/next of kin for the publication of any potentially identifiable images or data included in this article.

## Author contributions

AC, CR, and RB conducted the experiments and interpreted the data; AC, RB, SR, AL, and LV contributed to MV analysis by flow cytometry; AC, CR, RB, LA, ML, RM, YK, and ET contributed to MV production and ELISA experiment realization; CF, KR, A-LJ, T-AT, RL, FD-G, and GK contributed to sample collection and analysis of clinical data; FD-G handled funding of the manuscript; RL made a critical revision of the manuscript; GK took care of patients and handled funding and supervision of the project, helped with the interpretation of the experiments, and wrote the manuscript with AC. All authors contributed to the article and approved the submitted version.

## Glossary

**Table:**

IL	interleukin
JIA	juvenile idiopathic arthritis
sJIA	systemic juvenile idiopathic arthritis
MVs	microvesicles
PBMCs	peripheral blood mononuclear cells
THP-1	Tohoku Hospital Pediatrics-1
PMA	phorbol 12-myristate 13-acetate
NLRP3	nucleotide-binding domain
P2X7R	P2X purinoceptor 7
TF	tissue factor
ELISA	enzyme-linked immunoassay
HUVECs	human umbilical vein endothelial cells
LPS	lipopolysaccharide
ATP	adenosine triphosphate
kDa	kilodaltons
TLR	toll-like receptors
NLR	nucleotide-binding oligomerization domain (NOD)-like receptors ASC, apoptosis-associated speck-like protein containing a CARDS
CARD	caspase recruitment domain
HLA	human leukocyte antigen
RNA	ribonucleic acid
ILAR	International League of Associations for Rheumatology
JADAS10	Juvenile Arthritis Disease Activity Score 10
PPP	platelet-poor plasma
FITC	fluorescein isothiocyanate
CD	cluster of differentiation
PC7	phycoerythrin cyanine 7
APC	allophycocyanin
Ab	antibody
PBS	phosphate-buffered saline
IgG	immunoglobulin G
ICAM-1	intercellular adhesion molecule 1/CD54
VCAM-1	vascular cell adhesion protein 1/CD106
HEPES	hydroxyethylpiperazine ethane sulfonic acid
BSA	bovine serum albumin
IL-1Ra	IL-1 receptor antagonist
ANA	antinuclear antibody
CRP	C-reactive protein
ESR	erythrocyte sedimentation rate
MISEV	Minimal Information for the Study of Extracellular Vesicles
CTRL	control
YVAD	acetyl-tyrosyl-valyl-alanyl-aspartyl
DNA	deoxyribonucleic acid
Ca^2+^	calcium
TEM	transmission electron microscopy
CINC-1	cytokine-induced neutrophil chemoattractant-1
HC	healthy control
GADPH	glyceraldehyde-3-phosphate dehydrogenase
polyA	polyarticular
oligoA	oligoarticular
F	feminine
M	male
HG	hemogram
CS	corticosteroids
DMARDS	disease-modifying antirheumatic drugs
RIPA	radio-immunoprecipitation assay buffer
