# Supplementary material for: Macrophage IL-1β-positive microvesicles exhibit thrombo-inflammatory properties and are detectable in patients with active juvenile idiopathic arthritis
Source: Front Immunol. 2023 Nov 21;14:1228122. doi: 10.3389/fimmu.2023.1228122 (PMC10703381; doi:10.3389/fimmu.2023.1228122)
Supplement: Supplementary file 1 [file DataSheet_1.pdf]

frontiers

Supplementary Material

Macrophage IL-1β-positive-microvesicles exhibit thrombo-inflammatory properties and are detectable in patients with active Juvenile-Idiopathic-Arthritis

Audrey Cambon[1, 2*], Charlotte Rebelle[1, 3*], Richard Bachelier[1], Laurent Arnaud[4], Stéphane Robert[1], Marie Lagarde[1], Romain Muller[1,5], Edwige Tellier[1], Yéter Kara[1], Aurélie Leroyer[1], Catherine Farnarier[6], Loris Vallier[1], Corinne Chareyre[1], Karine Retornaz[3], Anne-Laure Jurquet[3], Tu-Anh Tran[7], Romaric Lacroix[1,8], Françoise Dignat-George[1,8], and Gilles Kaplanski[1,5°]

[1.] Aix-Marseille Université, INSERM, INRAE, C2VN, Marseille, France

[2.] Service de Médecine interne et d'Infectiologie, HIA Sainte-Anne, Toulon, SSA, France.

[3.] Service de Pédiatrie, AP-HM, Hôpital Nord, Marseille, France

[4.] Laboratoire d'Hématologie, AP-HM, La Timone, Marseille, France

[5.] Service de Médecine interne et d'Immunologie clinique, AP-HM, La Conception, Marseille, France.

[6.] Laboratoire d'Immunologie, AP-HM, La Conception, Marseille, France

[7.] Service de Pédiatrie, CHU Nîmes, Hôpital Carémeau, Nîmes, France

[8.] Service d'Hématologie et de biologie vasculaire, AP-HM, Marseille, France

*** Audrey Cambon and Charlotte Rebelle have equally participated to this study**

°Correspondence:
Gilles Kaplanski, C2VN, Faculté de Pharmacie, 27 Bd J. Moulin, 13005 Marseille, France.
gilles.kaplanski@ap-hm.fr

Keywords: microvesicles, IL-1β, NLRP3-inflammasome, tissue factor, juvenile-idiopathic-arthritis

1 Supplementary methods

Cell culture

The human monocytic leukaemia cell line THP-1 (THP-1 Cell Line human, Leukemic monocyte, 88081201, Sigma Aldrich, France) was cultured in RPMI 1640 medium supplemented with

1% Penicillin-streptomycin, 1% L-Glutamine, 1% sodium pyruvate (Invitrogen Life Technologies, UK) and 10% foetal calf serum. THP-1 cells were differentiated into macrophages in presence of 50ng/ml PMA (phorbolmyristate acetate, Sigma Aldrich, France) for 24 hours. Peripheral blood mononuclear cells (PBMCs) were isolated from heparinized peripheral blood of healthy donors by density gradient centrifugation on Lymphocyte Separation Medium LSM1077 (GE Healthcare, USA) and then differentiated into macrophages by culturing with 100ng/ml GM-CSF (Genzyme Corporation, USA) for 7 days. Cells were stimulated overnight with 10µg/ml LPS (Escherichia coli serotype O26:B6, Sigma Aldrich, France), followed by adjunction of 1mM ATP (Abcam, UK) for 1 hour. In some experiments, 10 µM of caspase-1 inhibitor YVAD (Sigma Aldrich, France) was added 3 hours prior to LPS. HUVECs (Human Umbilical Vein Endothelial Cells) were isolated from the umbilical vein of neonates issued from normal pregnancies. The local ethics committee of the Marseille Public Hospitals approved this collection and all the parents gave written informed consent for the use of cord blood cells in accordance with the Declaration of Helsinki. HUVECs were obtained as previously described(1), cultured in complete EGM-2 medium reconstituted as indicated by the manufacturer (Lonza, USA), and used between passages 3 to 5.

Extracellular vesicles isolation and purification

The culture medium supernatant was centrifuged for 5 min at 300g to pellet cell and at 4500g for 20 min to remove apoptotic bodies. Purified MV were concentrated by ultrafiltration (three times at 70 000g 90 min) and stored at −80°C in PBS-/-, after enumeration by flow cytometry, using Annexin V positive staining, according to Cointe S. et al. (28).

-) MV gating strategy and staining protocol

MV analysis by flow cytometry was performed using a 4-laser (Violet–Blue–Yellow–Red) CytoFLEX S cytometer (Beckman Coulter) provided with a plate reader. Instrument performances were checked daily using CytoFLEX Daily QC fluorosphere beads and SPHERO Rainbow 8-peak (Spherotech, Lake Forest, USA). The stability of the large MV scatter gate was monitored by the use of Megamix-Plus Forward Scatter (FSC) and Side Scatter (SSC) purchased from BioCytex (Marseille, France). The gating strategy is illustrated in Supplemental Figure S1.

Regarding the staining step, all antibodies were centrifuged for 2 min at 13,000 g to remove aggregates prior to use. MV (2µL) were incubated with 2µL of FcR blocking reagent (Miltenyi Biotec, ref 130-

059-90110), 10µL of annexin-V-FITC (AnnV) (Beckman Coulter), 2µL of CD18 PE or control isotype antibody and 20µL of annexin-V binding buffer. After 20 min of incubation at room temperature (RT), samples were diluted in 200µL of annexin-V binding buffer and analyzed. To prevent carryover issues, a washing well was performed between each sample.

-) Tunable resistive pulse sensing

Tunable resistive pulse sensing (TRPS) was performed using a qNano Gold TRPS measurement instrument (Izon, Oxford, UK). Before using the samples, the instrument was calibrated with CPC200 beads (mean diameter 210nm). NP150 nanopore membranes stretched at approximately 41nm were used. Voltage was set in 0.36V to achieve a stable current 110–1120 nA and pressure at 12 mbars, with root mean square noise below 10 pA. Samples and calibration beads were diluted in running buffer (PBS filtered under 0.22 µm). Samples were analyzed using an NP150 nanopore (Izon). Measurement and analysis were performed with Izon Control Suite 3.2 Software.

-) Western blot

Western blot was performed on MV pellets obtained after three ultracentrifugations at 70,000g for 90 min. MV pellets were lysed with RIPA buffer, next, separated on a 4%–12% NUPAGE gel under SDS or no-reducing conditions (for CD81) and then, transferred onto nitrocellulose membranes (Amersham Protran, Merck Sigma–Aldrich, St Quentin Fallavier, France). Membranes were blocked with 3% BSA/tris-buffered saline (TBS, ET220B, Euromedex, Souffelweyersheim, France) for 1h at RT. Then, the membranes were incubated overnight at 4°C with antibodies against the designated antigens and purchased from Cell Signaling Technology, integrin β3 (1:1000, Cell Signaling Technology Cat# 4702, RRID:AB_2296320) and glyceraldehyde-3 phosphate dehydrogenase (GAPDH) (1:1000, Cell Signaling Technology Cat# 2118, RRID:AB_561053), from Thermo Fisher Scientific CD63 (1:1000, Thermo Fisher Scientific Cat# 10628D, RRID:AB_2532983) and albumin (1:1000, Thermo Fisher Scientific Cat# MA5-32531, RRID:AB_2809808). Next, horseradish peroxidase (HRP)-conjugated secondary antibody (1:5000, Thermo Fisher Scientific Cat# 31430, RRID:AB_228307) was added for 1h at RT. Immunocomplexes were visualized by enhanced chemiluminescence (ECL) according to the manufacturer's instructions (Pierce, Rockford, IL, USA). Specific bands were detected using a G-BOX Imaging System (GeneSys, Cambridge, UK).

Animal Studies

The local ethical committee approved animal studies. C57BL/6J mice received one intraperitoneal injection of PBS or 30mg/kg IL-1Ra (anakinra, Swedish Orphan Biovitrum AB, France). They were then injected in peritoneal cavity with either 25×10^6 MV resuspended in PBS either PBS alone. Fifteen hours after injections, blood was collected in EDTA tubes by intra-cardiac puncture (from anesthetized mice with 2% isoflurane) and plasmas were separated by centrifugation before cytokine measurements. After blood collection, animals were immediately killed by cervical discolcation and peritoneal cavities were flushed with 1.25mM PBS-EDTA. The peritoneal fluid was harvested and cells were pelleted by centrifugation. Then, cells were suspended and incubated with 0.5µg/ml anti-Fc-receptor antibody (2.4 G2, BD Biosciences, France) and labelled with the following mix of antibodies diluted in PBS: Ly6c-FITC (1:400, BioLegend Cat# 128005, RRID:AB_1186134), F4/80-PE (1:400, BioLegend Cat# 111703, RRID:AB_2936728), Gr1 PerCP-Cy5.5 (1:1000, BioLegend Cat# 108427, RRID:AB_893561)), CD11c PE-Cy7 (1:800, BioLegend Cat# 117317, RRID:AB_493569), Mac1-APC (1:1000, BioLegend Cat# 101211, RRID:AB_312794), CD45 eFluor 780 (1:1000, Thermo Fisher Scientific Cat# 47-0451-82, RRID:AB_1548781). After red blood cell lysis by addition of 500µl ACK lysing buffer (ThermoFisher Scientific, USA) for 10 minutes at room temperature, the total number of leukocytes, neutrophils, or monocytes in the peritoneal exudate was determined by counting CD45+, CD45+/Gr1 high/F4/80 low or CD45+/Gr1 low/F4/80 high cells respectively, using a Gallios flow cytometer and Fluorescent Flowcount beads (Beckman Coulter, France).
