# Supplementary figures and images for: Macrophage IL-1β-positive microvesicles exhibit thrombo-inflammatory properties and are detectable in patients with active juvenile idiopathic arthritis
Source: Front Immunol. 2023 Nov 21;14:1228122. doi: 10.3389/fimmu.2023.1228122 (PMC10703381; doi:10.3389/fimmu.2023.1228122)

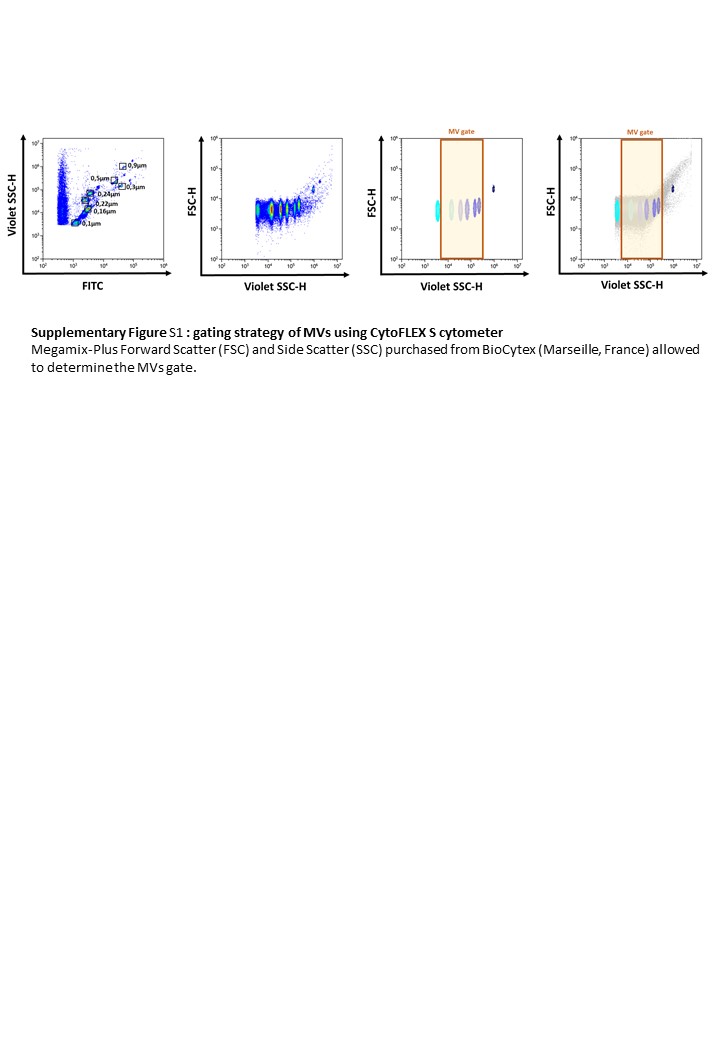

Supplement: Supplementary file 2 [file Image_1.jpeg]

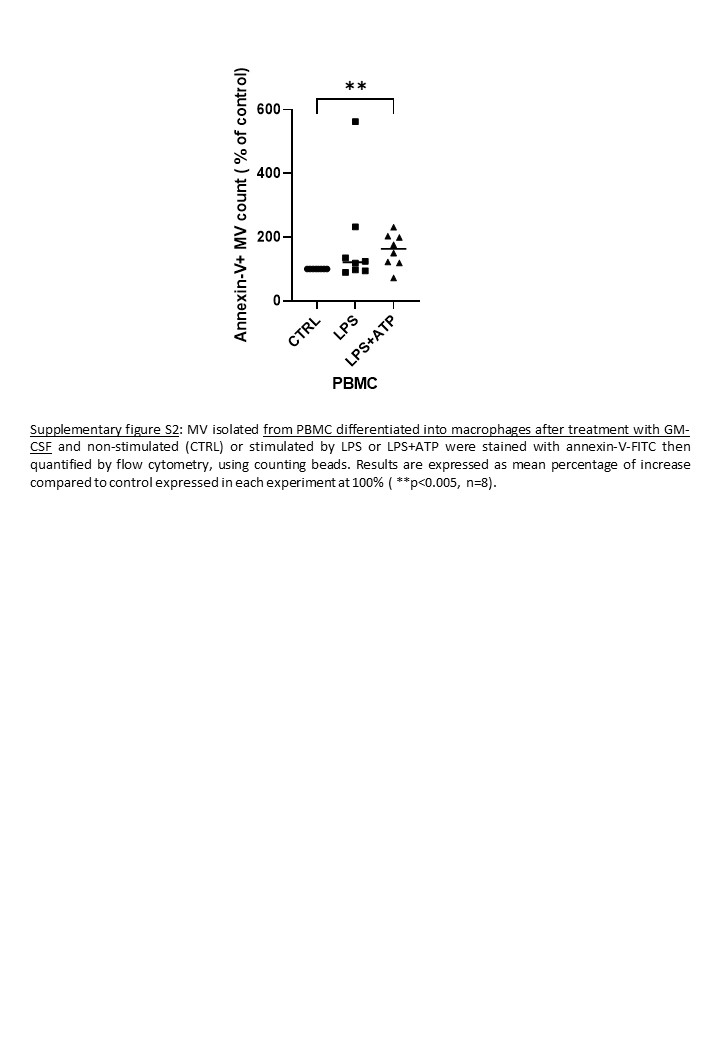

Supplement: Supplementary file 3 [file Image_2.jpeg]

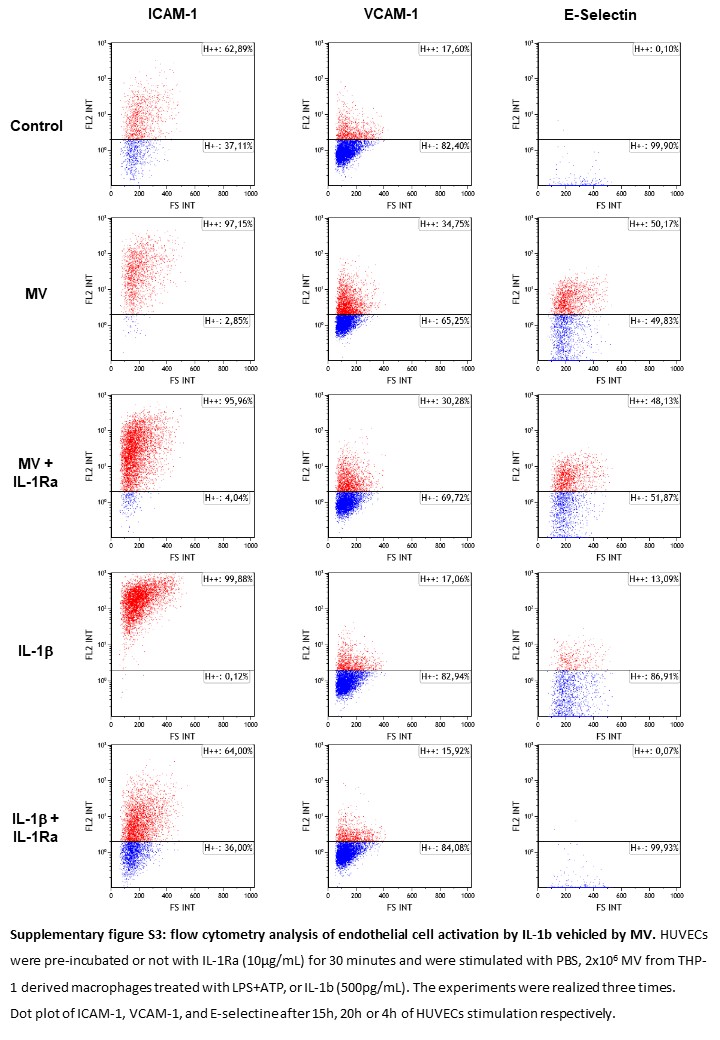

Supplement: Supplementary file 4 [file Image_3.jpeg]

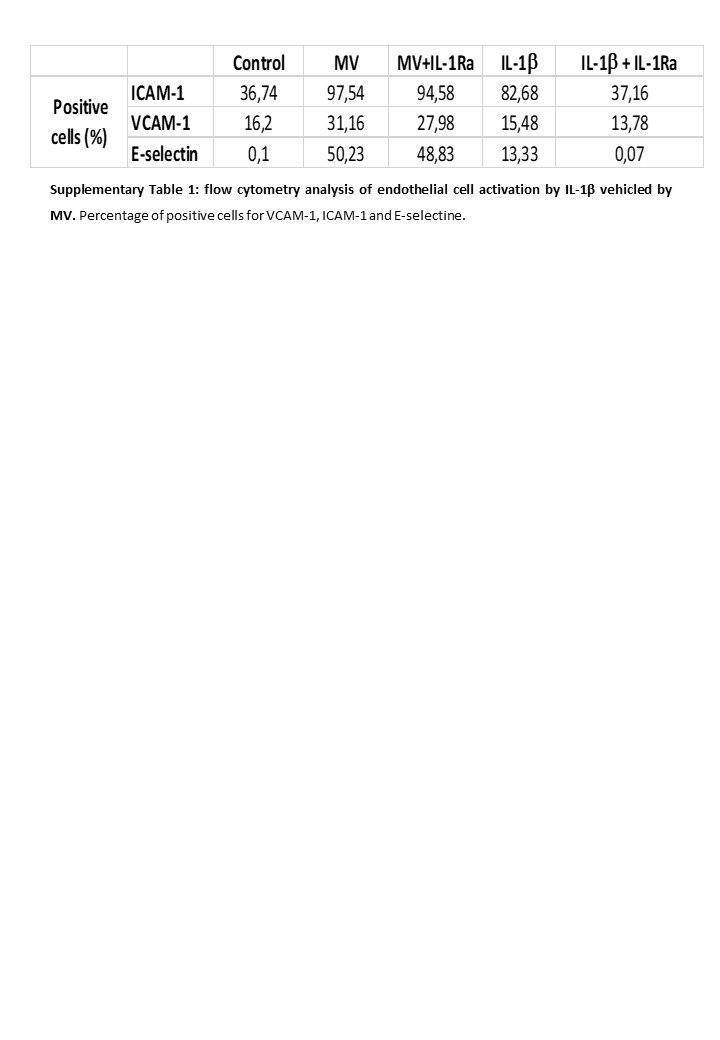

Supplement: Supplementary file 5 [file Image_4.jpeg]
